# Non-*Saccharomyces* yeast derivatives: Characterization of novel potential bio-adjuvants for the winemaking process

**DOI:** 10.1016/j.crfs.2024.100774

**Published:** 2024-05-22

**Authors:** Valentina Civa, Fabio Chinnici, Gianluca Picariello, Emma Tarabusi, Matteo Bosaro, Ilaria Mannazzu, Paola Domizio

**Affiliations:** a Department of Agriculture, Food, Environment and Forestry (DAGRI), University of Florence, Italy; b Department of Agricultural and Food Sciences, University of Bologna, Italy; c Istituto di Scienze dell’Alimentazione - CNR, Via Roma 52 A/C, I-83100, Avellino, Italy; d Italiana Biotecnologie, Via Vigazzolo 112, 36054, Montebello Vicentino, Italy; e Department of Agricultural Sciences, University of Sassari, Sassari, Italy

**Keywords:** Inactivated dry yeast, Polysaccharide, Mannoprotein, Glutathione, Lipid, Oxygen consumption

## Abstract

Winemakers have access to a diverse range of commercially available Inactivated Dry Yeast Based products (IDYB) from various companies and brand names. Among these, thermally inactivated dried yeasts (TIYs) are utilized as yeast nutrients during alcoholic fermentation, aiding in the rehydration of active dry yeasts and reducing ochratoxin A levels during wine maturation and clarification. While IDYB products are generally derived from *Saccharomyces* spp., this study investigates into the biodiversity of those deriving from non-*Saccharomyces* for potential applications in winemaking. For that *S. cerevisiae* and non-*Saccharomyces* TIYs were produced, characterized for nitrogen and lipid content using FT-NIR spectroscopy, and applied in a wine-like solution (WLS) for analyzing and quantifying released soluble compounds. The impact of TIYs on oxygen consumption was also assessed. Non-*Saccharomyces* TIYs exhibited significant diversity in terms of cell lipid composition, and amount, composition, and molecular weight of polysaccharides. Compared to that of *S. cerevisiae*, non-*Saccharomyces* TIYs released notably higher protein amounts and nHPLC-MS/MS-based shotgun proteomics highlighted the release of cytosolic proteins, as expected due to cell disruption during inactivation, along with the presence of high molecular weight cell wall mannoproteins. Evaluation of antioxidant activity and oxygen consumption demonstrated significant differences among TIYs, as well as variations in GSH and thiol contents. The Principal Component Analysis (PCA) results suggest that oxygen consumption is more closely linked to the lipid fraction rather than the glutathione (GSH) content in the TIYs. Overall, these findings imply that the observed biodiversity of TIYs could have a significant impact on achieving specific oenological objectives.

## Highlights

• Yeast biodiversity impact on TIYs properties in wine like solution.
• TIYs deriving from different species differ for antioxidant activity and oxygen consumption.
• Oxygen consumption seems related to content and composition of TIYs lipid fraction.
• TIYs biodiversity is functional to achieve specific oenological objectives.

## Graphical abstract

**Figure:**
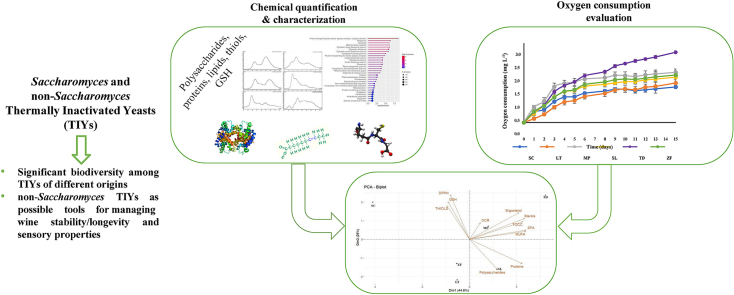

## 1 Introduction

The development of innovative solutions and biotechnological tools aimed at ensuring the final quality of wine and responding to the increasing interest of consumers in environmental and health-related issues is a hot topic for the wine industry. Among the possible biotechnological tools, Inactive Dry-Yeast Based products (IDYB) (López-Solís et al., 2017) are attracting attention. IDYB currently on the market and authorized by the Organization International de la Vigne et du vin (OIV) are derived from *Saccharomyces cerevisiae*. These include: (i) yeast inactivated by physical treatments followed by drying; (ii) yeast lysates for which a partial spontaneous or induced enzymatic degradation of the cell content is followed by drying; (iii) yeast hulls consisting of the insoluble cell components deriving from yeast walls; (iv) yeast protein extracts that mainly contain the cytoplasmic cell protein fraction.

Indeed, IDYB characteristics are highly variable and depend on different factors. In addition to the techniques used for inactivation, the yeast strain employed, the culture conditions (Guilloux-Benatier and Chassagne, 2003) and the growth stage have a marked effect on IDYB composition (López-Solís et al., 2017; Pozo-Bayón et al., 2009a). Thus, based on the oenological objective, winemakers can choose from a wide variety of commercial IDYB, provided by different companies under various brand names. IDYB can be used to manage the fermentation for several purposes: to enhance yeast and lactic bacteria growth, thereby preventing stuck or sluggish fermentations; for wine fining to modulate wine astringency and/or adsorb toxic compounds and other undesirable components, such ochratoxin A (OTA), octanoic and decanoic acids, 4-ethylphenol, geosmin, and some pesticides often used in vineyards; and for wine stabilization to improve wine colloidal or oxidative stability and wine sensory characteristics (Alexandre, et al., 1997; Andújar-Ortiz, et al., 2014; Comuzzo et al., 2011, 2017; Lubbers et al., 1994; Petruzzi et al., 2015; Pozo-Bayón, et al., 2009a; Pradelles et al., 2010). Yet, despite the growing interest by the oenological sector in these products, little is known on their actual composition and action mechanisms, causing great uncertainty among winemakers about the choice and use of suited products. For instance, in the utilization of yeast lysates as fermentation enhancers, the choice of the most appropriate commercial preparation should rely on the expected effect. In fact, while their “direct effect” on microbial growth is based on the percentage of soluble yeast cell compounds and the accessibility of micronutrients, their “indirect effect” depends on the availability of the insoluble fraction involved in the adsorption of toxic compounds (Pozo-Bayón et al., 2009b). Additionally, the application of yeast protein extracts for wine fining may decrease the efficacy of tannin-wine protein coupling if they contain mannoproteins (Lochbühler et al., 2015).

Thermal inactivation is the easiest way to obtain IDYB on an industrial scale. Thermally inactivated dried yeasts (TIYs) still maintain their cell content, although cell integrity is impaired (Bzducha-Wròbel et al., 2014; Rigou et al., 2021). According to Resolution OIV-OENO 459–2013, TIYs are claimed to be used as yeast nutrients at the beginning and during alcoholic fermentation, to promote the rehydration of active dry yeasts and to reduce ochratoxin A level during wine maturation and clarification. Besides, if needed, TIYs can be easily removed from wine through filtration.

Non-*Saccharomyces* yeasts, due to their biocontrol activity in pre-fermentative stages and during wine conservation (Simonin et al., 2018; Windholtz et al., 2021), the release of polysaccharides able to protect wine from protein haze (Domizio et al., 2014; Snyman et al., 2021) and their general impact on wine quality (Belda et al., 2017; Ciani et al., 2010; Jolly et al., 2014), may represent a promising alternative to conventional processing aids. For that they are currently proposed as biotechnological tools for the achievement of specific oenological objectives (Zara and Nardi, 2021). These yeasts differ in their enzymatic activities, fermentative properties, and outcomes on the final wine (Ciani et al., 2010). Possibly due to differences in cell wall composition (Ballou, 1976; Lozančić et al., 2021), they may release significantly higher amounts of polysaccharides in respect to *Saccharomyces* (Domizio et al., 2014), with possible different functional effects on the aggregation of tannins and protein stabilization.

With the aim of exploiting the wide biodiversity existing among yeasts of oenological interest in function of their possible impact on wine quality, here, non-*Saccharomyces* TIYs were produced and used in wine like solution (WLS). Soluble compounds released in WLS by *S. cerevisiae* and non-*Saccharomyces* TIYs, such as thiol compounds and polysaccharides, were quantified and characterized. Untargeted proteomic characterization of the released compounds was also performed to highlight differences among TIYs and evaluate their possible involvement in the wine colloidal stability. TIYs were also added in a model solution under catalytic conditions for evaluating the oxygen consumption.

## 2 Materials and methods

### 2.1 Chemicals

Glucose, fructose, mannose, sodium chloride (NaCl), mannan from *S. cerevisiae*, 4,4′-dithiodipyridine (DTDP), reduced glutathione (GSH), sodium acetate, sodium phosphate dibasic, copper (II) sulfate pentahydrate, iron (III) chloride hexahydrate, 3-mercaptopropionic acid (3MPA), bovine serum albumin (BSA), Schiff's reagent, *p*-benzoquinone, pullulan molecular weight standards (96,351–1 KT), guanidine HCl, trichloroacetic acid (TCA), trifluoroacetic acid (TFA), dithiothreitol (DTT), iodoacetamide (IAA) were provided from Sigma-Aldrich (Milan, Italy). Absolute ethanol, methanol, acetone, LC-MS grade water and acetonitrile, ammonium formate and ethylenediaminetetraacetic acid (EDTA) were provided from Carlo Erba Reagents (Milan, Italy). Citric acid and L-(+)-tartaric acid were provided from JT Baker (Phillipsburg, NJ, USA). Chloridric acid (HCl) was provided from Honeywell Fluka (Seelze, Germany). Trolox and 1,1-diphenyl-2-picryl-hydrazyl free radical (DPPH•) were provided from Thermo Fisher Scientific (USA). Yeast extract was provided from Oxoid (Milan, Italy). Pancreatic peptone was provided from VWR Chemicals (USA). Coomassie Brilliant Blue G-250 and Bio-safe Coomassie G-250 dye reagent was provided by Bio-Rad (Laboratories, Hercules, CA, USA).

### 2.2 Yeast strains

Five non-*Saccharomyces* yeast strains belonging to the culture collection of the Department of Agriculture, Food, Environment and Forestry (DAGRI University of Florence) were used. A commercial starter, Lalvin EC1118 (Lallemand Inc., Montreal, Canada), was used as reference strain for *S. cerevisiae* and for analytical comparison (Table 1).

**Table 1:** Origin of yeast strains.

Strain	Species	Origin
# EC1118	*Saccharomyces cerevisiae*	Lallemand^a^
# 42	*Zygosaccharomyces florentinus*	DAGRI^b^
# 46	*Metschnikowia pulcherrima*	DAGRI^b^
# 64	*Saccharomycodes ludwigii*	DAGRI^b^
# 92	*Torulaspora delbrueckii*	DAGRI^b^
# 101	*Lachancea thermotolerans*	DAGRI^b^

a Lallemand Inc. (Montreal, Canada).

b Department of Agriculture, Food, Environment and Forestry (DAGRI), University of Florence, Italy.

### 2.3 Thermally inactivated yeasts (TIYs) preparation and utilization

Yeast pre-cultures were grown in 100 mL flasks containing 75 mL of a growth medium (2.5 % yeast extract, 2 % peptone, 5 % glucose, 5% fructose) at 27 °C in an orbital shaker at 150 rpm. After 24 h, 1 % of each preculture was inoculated in flasks containing 750 mL of the same medium and incubated for 72 h as above indicated. The cultures were then centrifuged at 4 °C for 8 min at 8000 rpm, the cell pellets were thoroughly washed and resuspended in sterile distilled water with a 1:5 (w/v) biomass water ratio. Thermal inactivation was carried out at 121 °C for 1 h and the inactivated yeast biomass was freeze-dried to obtain thermally inactivated yeast powder (TIY).

**Fig. 1:**
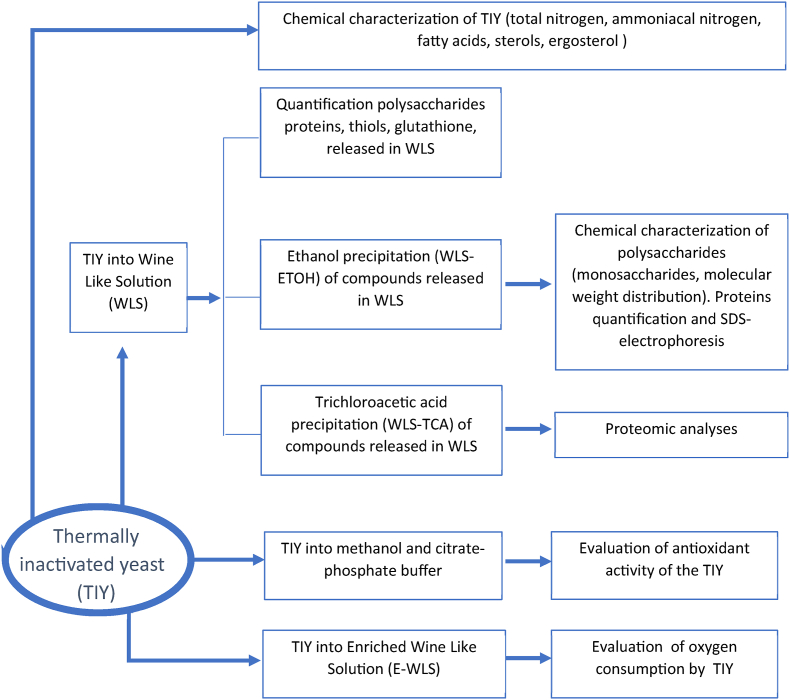
Experimental workflow.

In Fig. 1 is reported the experimental workflow adopted. In detail, 4 mg mL^−1^ of each TIY was resuspended in WLS (WLS: ethanol 12 %; tartaric acid 0.03 M; pH 3.2) and kept in an orbital shaker (80 rpm) at room temperature. After 48 h, the samples were centrifuged (8 min at 6000 rpm) and WLS supernatants (WLS-S) were filtered through a 0.45 μm acetate cellulose filter. When required, polysaccharides and proteins in WLS-S were concentrated by ethanol precipitation (WLS-ETOH) or by trichloroacetic acid precipitation (WLS-TCA) in acetone.

### 2.4 Chemical characterization of TIYs

Each TIY was characterized by Fourier transform near-infrared (FT-NIR) diffuse reflectance spectroscopy. FT-NIR spectra were obtained using a PerkinElmer FRONTIER FT-NIR spectrometer (Waltham, MA) with NIRA Sample Spinner. The samples, in dry-powder form, were analyzed without further preparation by placing them in a glass Petri dish directly on the integrating sphere and measuring through the bottoms of the vessels. Each yeast derivative was scanned twice in the 10,000-4000 cm^−1^ wavelength range. Spectral data were acquired using the Spectrum IR software (PerkinElmer). Each acquisition was the combination of 32 scans to reduce S/N ratio. The quantification was done using the Spectrum Quant software (PerkinElmer) with the algorithm QuantPlusPLS1. The pre-processing of the spectra was Derivative First Order. Accuracy, precision, and robustness of the method were determined using 125 samples in a validation set. Each parameter used for the validation set was calculated with the official OIV wet chemistry method.

### 2.5 Quantification, purification and characterization of polysaccharides

Polysaccharides released by each TIY in the WLS-S were quantified by high-performance liquid chromatography (HPLC), according to the method reported in Millarini et al. (2020). Briefly, 20 μL of each sample were injected into the HPLC apparatus (Varian Inc., Palo Alto, CA, USA) equipped with a 410 series autosampler, a 210 series pump, and a 356-LC refractive index (RI) detector. Isocratic separation was performed on a TSK Gel-OLIGO-PW (808,031) column (30 cm × 7.8 mm i. d.) and a TSK-GEL OLIGO (808,034) guard column (4 cm × 6 mm i. d.) (Supelco, Bellefonte, PA, USA). The mobile phase was 0.2 M NaCl, at a flow rate of 0.8 mL min^−1^. Peaks were quantified by comparison with an external calibration curve built with mannan (Sigma-Aldrich, Milan, Italy) at concentration from 50 to 1000 mg L^−1^. Peaks were integrated using the software Galaxie Chromatography Data System (version 1.9.302.530) (Varian Inc., Palo Alto, CA, USA). All the analyses were carried out in duplicate.

The monosaccharide composition of polysaccharides was determined after ethanol precipitation of WLS-S fraction. In detail, four volumes of cold 95 % ethanol containing 0.3 M HCl was added to the WLS-S fraction and then kept under 4 °C for 24 h. The precipitated fraction was then separated by centrifugation (8000 g, 4 °C, 30 min). The supernatants were discarded, and the pellets were washed two times with four volumes of 95 % of cold ethanol and freeze dried. Finally, the freeze-dried pellet, that is WLS-ETOH, was resuspended at the concentration of 1 mg mL^−1^ in 2 N TFA. This solution was then heated at 120 °C for 120 min. TFA was then removed using a rotary evaporator and the dried extract was re-solubilized in deionized water and analyzed using the same HPLC system described before. Injection volume was 25 μL. Isocratic separation was performed at 70 °C on a 300 × 7.7 mm PL Hi-Plex Pb 8 μm column (Agilent Technologies, Palo Alto, CA, USA). The mobile phase was MilliQ water at a flow rate of 0.6 mL min^−1^. Glucose and mannose were quantified using calibration curves built in the 0.008–0.50 mg L^−1^ and 0.25–2 mg L^−1^ ranges, respectively. All the hydrolysis were performed in triplicate.

Molecular weight distribution of polysaccharides was determined according to the method reported in Fanzone et al. (2012), with minor modifications. Briefly, the dried pellet WLS-ETOH was resuspended in 30 mM ammonium formate at the concentration of 2 mg mL^−1^. After that, 50 μL was injected onto the column. The analyses were performed using the same HPLC apparatus as above reported. Separation was carried out at room temperature on two Shodex Ohpak SB-803 and SB-804 HQ columns connected in series (300 mm × 8 mm I.D.; Showa Denko, Japan), and the temperature of cell RID was 35 °C. The mobile phase was the same used to resuspend the WLS-ETOH fraction, at a flow rate of 0.6 mL min^−1^ for 60 min. Calibration was performed with narrow pullulan molecular weight standards from 342 to 805,000 Da (Sigma-Aldrich, Milan, Italy).

### 2.6 Protein quantification

Proteins in WLS-ETOH fraction were quantified by dye-binding Bradford assay (Bradford 1976). Briefly: 20 μL of WLS-ETOH suspension (1 mg in 120 μL of water) were added to 1 mL of Coomassie Brilliant Blue G250 (Bio-Rad), diluted at a ratio of 1:4. Protein concentration was calculated using a calibration curve obtained with Bovine Serum Albumin (BSA) as the external standard.

### 2.7 Proteomics and biocomputational analysis

For proteomic analyses, the protein fraction from 5 mL aliquots of TIYs WLS-S was precipitated overnight at −20 °C with 30 mL of 10 % (w/v) TCA in acetone. The protein pellet (WLS-TCA) was recovered by centrifugation (8000 g, 4 °C, 30 min), washed three times with −20 °C cold acetone and lyophilized. Protein powders were dissolved in 1 mL of denaturing and reducing buffer (6 M guanidine HCl, 50 mM Tris, 1 mM EDTA, 10 mM DTT, pH 8.0) and incubated at 56 °C for 1 h under N2. After reduction, cysteines were alkylated with 55 mM IAA for 30 min at room temperature in the dark.

For N-deglycosylation with peptide N-glycosidase F (PNGase F) Cys-alkylated proteins were desalted using prepacked G-25 EconoPak 10 DG columns (Bio-Rad, Milan, Italy), eluting with 50 mM ammonium bicarbonate, pH 7.8. After quantification with the modified micro-Lowry assay (kit from Sigma-Aldrich), 100 μg of protein for each yeast strain were diluted in an equal volume of a 0.2 % (w/v) RapiGest SF (Waters, Milford, MA, USA) solution prepared in 50 mM ammonium bicarbonate, pH 7.8 and incubated 6 h at 37 °C with 2 U of PNGase F from *Flavobacterium meningosepticum* (Boehringer Mannheim, Germany). After enzymatic N-deglycosylation, the RapiGest SF was inactivated with 15 μL of 0.5 N HCl and after centrifugation (10,000 g, 4 °C, 30 min) the clear supernatants were transferred in clean tubes and lyophilized. Cys-alkylated and N-deglycosylated proteins were sequentially digested in 100 μL of 50 mM ammonium bicarbonate, pH 7.8, with 1/100 sequencing grade Lys-C, overnight at 37 °C and with 1/50 sequencing grade modified trypsin (enzymes from Promega, Madison, WI, USA) overnight at 37 °C. Tryptic peptides were purified by solid-phase extraction with C 18 pre-packed spin columns (Pierce/Thermo Fisher Scientifics, Rockford, IL, USA), washing extensively with 0.1 % (v/v) TFA and eluting with 50 μL of 70 % acetonitrile (0.1 %) (v/v) TFA. Peptides were vacuum dried, lyophilized and re-constituted in 0.1 % formic acid for analysis.

Nanoflow-high performance liquid chromatography-electrospray tandem mass spectrometry (nHPLC-MS/MS) analyses were performed using an Ultimate 3000 cromatographer (Dionex/Thermo Scientific, San Jose, CA) coupled with a Q Exactive Orbitrap mass spectrometer (Thermo Scientific). Nearly 2 μg of each peptide pool was loaded through Acclaim PepMap 100 trap columns (75-μm i. d. × 2 cm; Thermo Scientific) through the autosampler (Thermo Scientific). Eluent A and B were 0.1 % formic acid (v/v) in LC – MS grade water and 0.1 % formic acid (v/v) in 80 % aqueous acetonitrile, respectively. Peptides were separated using an EASY-Spray™ PepMap C18 column (25 cm × 75 μm) with 2 μm particles and 100 Å pore size (Thermo Scientific), applying a 2–50 % gradient of B over 120 min after 12 min of isocratic elution at 2% B, at a constant flow rate of 300 nL min-1. MS1 precursor spectra were acquired in the positive ionization mode using the following instrumental parameters: acquisition range: 1600–300 m/z; resolving power: 70,000 full width at half maximum (FWHM); automatic gain control (AGC) target: 1 × 106 ions; maximum ion injection time: 100 ms. The spectrometer operated in full scan MS1 and Top-10 data-dependent acquisition mode, applying a 12-s dynamic exclusion. MS/MS spectra were obtained at a resolving power of 17,500 FWHM. Ions with charge +1 or greater than +6 were excluded from the MS/MS fragmentation. Spectra were elaborated using the software Xcalibur version 3.1 (Thermo Scientific). For each yeast strain, proteolytic peptides were analyzed in triplicate, alternating the acquisition with the analysis of blank samples.

For the bioinformatic and computational proteomic analyses, a preliminary identification of the protein components was carried out with the Protein Prospector Batch-Tag Web tool (https://prospector2.ucsf.edu), using the mgf files generated from the LC-MS runs with the MS Convert tool of the open-source ProteoWizard 3.0 software (https://proteowizard.sourceforge.net/). Searches in the UniProtKB database were taxonomically restricted to microorganisms and then further refined by searching against yeasts. Afterwards, protein identifications were validated using the Proteome Discoverer software vers. 2.1 (Thermo Scientific) based on the Sequest algorithm. In this case, raw spectra were searched against species-specific protein/genomic databases downloaded from NCBI on March 2023. Searching parameters were: Cys-carbamidomethylation of a static modification; methionine (Met) oxidation, pyroglutamic acid at N-terminus glutamine (Gln), Gln and asparagine (Asn) deamidation, serine/threonine (Ser/Thr) phosphorylation, as variable modifications; mass tolerance value of 10 ppm for precursor ion and 15 Da for MS/MS fragments; trypsin as the proteolytic enzyme with missed cleavage up to 2. Protein identification scores were calculated by Target Decoy Peptide Spectrum Matches (PSMs) filtering working at a 1 % protein-level false discovery rate (FDR). LC-MS runs were also analyzed using the Mascot-MS search engine (https://www.matrixscience.com), restricting the taxonomy to Fungi in the curated SwissProt database, to obtain the emPAI (exponentially modified Protein Abundance Index) values. Gene Ontology (GO) enrichment and functional classification analysis were carried out using the list of filtered proteins identified with at least two unique peptides, using the web-based PANTHER (Protein Analysis Through Evolutionary Relationships) classification system (https://www.pantherdb.org, release 16.0) and the open source ShinyGO v0.75 for the *Saccharomyces cerevisiae* organism (https://bioinformatics.sdstate.edu/go/).

### 2.8 Gel electrophoresis

Precipitated proteins present in WLS-ETOH fraction were diluted with ultrapure MilliQ water and protein electrophoresis was performed by using 10% sodium dodecyl sulfate-polyacrylamide gel electrophoresis (SDS-PAGE) (Laemmli, 1970), as previously described (Millarini et al., 2020). Blue precision plus protein standard (Bio-Rad) was loaded. Bio-Safe Coomassie G-250 stain (Bio-Rad) and periodic acid Schiff's reagent (Sigma-Aldrich) were used to stain protein and glycoprotein bands, respectively.

### 2.9 Reduced glutathione (GSH)

GSH quantification was performed according to Tirelli et al. (2010) with minor modifications. Briefly, 100 μL of *p*-benzoquinone (864 mg L^−1^ in methanol) was added to 3 mL of WLS-S and vortexed for 1 min. Then, 1 mL of 3-mercaptopropionic acid (106 mg L^−1^ in citrate buffer, pH 3.5) was added and vortexed for 1 min. After filtration (0.45 μm regenerate cellulose filter), the samples were immediately analyzed in a HPLC instrument (Jasco, Tokyo, Japan) equipped with a quaternary gradient pump PU-2089, an autosampler AS-2057 Plus Intelligent Sampler and a UV/Vis MD-910 PDA detector, set at 303 nm. The column was a C18 Poroshell 120 (Agilent technologies), 2.7 μm, (4.6 × 150 mm), operating at 30 °C with a flow rate of 0.8 mL min^−1^. Elution solvents were 0.05 % trifluoroacetic acid in HPLC grade water (Eluent A) and acetonitrile (Eluent B). Gradient elution was as follows: from 98 to 83 % A in 12 min, 83 to 70 % A in 3 min, 70 to 20 % A in 6 min and finally to 98 % A in 2 min. Quantification was performed by means of calibration curves previously obtained by duplicate injections of pure standard solutions of GSH at concentrations ranging from 0.1 to 50 mg L^−1^, prepared in 50 mM of citrate buffer (pH 5.0) and derivatized as described above. All the analyses were carried out by triplicate.

### 2.10 Total thiols

Total thiols were determined using the DTDP method, as described by Gallardo-Chacón et al. (2010). Briefly, each TIY was resuspended in acetate buffer (pH 3.6, 0.3 M). After centrifugation, 300 μL of supernatant was mixed with acetate buffer (pH 4.5, 0.1 N) containing EDTA 0.2 mM to a final volume of 3 mL. Then, 125 μL of DTDP (4 mM in 12 mM HCl) was added and, after 5 min at room temperature, the samples were filtered through a PTFE 0.45 μm pore size filter. The absorbance of the supernatant was measured at 324 nm, and the amount of thiols was expressed in mg of reduced glutathione (GSH) per g of TIY, using a calibration curve prepared with GSH at concentration 0.06 mg L^−1^ up to1 mg L^−1^.

### 2.11 Antioxidant activity

The antioxidant activity of the TIY was evaluated using the DPPH assay, as reported by Romanet et al. (2019), with minor modification. In detail, TIY were put directly in contact with DPPH solution (2,2-Diphenyl-1-picrylhydrazyl dissolved in a mixture of methanol and citrate-phosphate buffer, pH 3.6) by following the QUENCHER approach (Gökmen et al., 2009) in order to evaluate the effects of the soluble compounds released in the media and of the insoluble compounds present on the cell walls. The assay was performed in amber bottles and placed on an orbital shaker. According to Romanet et al. (2019), an aliquot of the sample was filtered through a 0.45 μm PTFE filter after 240 min, and the absorbance was measured using a UV–Vis spectrometer at 525 nm (Varian Cary 1E UV/Visible Spectrophotometer). The antioxidant activity of each TIY was evaluated using the DPPH assay. The standard curve was prepared using Trolox at concentrations ranging from 0.1 mmol L^−1^ up to 1 mmol L^−1^. Data were expressed in mmol of Trolox per g of dried powder.

### 2.12 Oxygen consumption

The oxygen consumption by each TIY was evaluated following the method described by Pons-Mercadé et al. (2021), using clear bottles (200 mL) filled with enriched wine like solution (E-WLS) containing 3 mg L^−1^ of iron, in the form of iron (III) chloride hexahydrate, and 0.3 mg L^−1^ of copper in the form of copper (II) sulfate pentahydrate, and closed with a crown cap and *bidule*. The bottles were maintained at 20 °C ± 0.1 during 15 days. Oxygen was measured and the bottles were gently shaken once a day, every 24 h. The dissolved oxygen in each sample was measured with the NOMASense O2 P300 (Nomacorc, Thimister Clermont, Belgium) equipped with PSt3 sensors (Nomacorc, Thimister Clermont, Belgium), based on non-invasive oxy-luminescence technology.

### 2.13 Data analysis

Resulting data were subjected to two-way analysis of variance (ANOVA). Fisher's LSD post hoc test was used to determine the significant differences between group means (p-value = 0.05) The means and the standard deviation of the mean (mean ± SD) are also reported. The data were analyzed using the Statgraphics Centurion software (Ver.XV, StatPoint Technologies, Warren-ton, VA). The application of PCA has been carried out in R Studio software.

## 3 Results and discussion

### 3.1 Chemical composition of TIYs

The gross composition of the TIYs, produced as described in materials and methods (paragraph 2.3), was determined by FT-NIR, a widely used analytical tool in the food industry. TIYs were characterized by a total nitrogen content ranging from 7.6 to 8.9 %, and by an ammoniacal nitrogen content ranging from 0.23 to 0.28 % of the dry matter (Table 2). These values are in accordance with the limits, for inactivated yeast, reported in the Resolution OIV-OENO 459–2013 (total nitrogen content, expressed as element N, less than 10 % of the dry matter). A high percentage of raw proteins (total nitrogen multiplied by 6.25) (Patterson et al., 2023) was present in all TIYs. Lipid composition exhibited considerable variability among TIYs. Given that they were obtained under identical experimental conditions, the observed differences were likely attributable to species-specific differences in lipid metabolism as well as to their different growth stage at the time of the inactivation process. Regarding fatty acids, this variability was primarily ascribed to the differing proportions of monounsaturated fatty acids (MUFAs). In particular, SC-TIY exhibited a lower amount of MUFAs compared to non-*Saccharomyces* TIYs (Table 2). Regarding sterols, both SC-TIY and ZF-TIY displayed significantly lower content (p < 0.05) than other non-*Saccharomyces* TIYs, with MP-TIY exhibiting the highest sterols content. Significant differences in ergosterol content were observed among TIYs. Fermentation additives include fatty acids and sterols that play a pivotal role in enhancing membrane integrity and supporting fermentative activity (Belviso et al., 2004; Varela et al., 2012). In particular, in the case of *S. cerevisiae* the addition of fermentation additives including ergosterol and unsaturated fatty acids may result in the enhancement of yeast stress resistance and ethanol tolerance (Landolfo et al., 2010; Casu et al., 2016; Ochando et al., 2017; Fairbairn et al., 2019) and influences the production of specific volatile compounds during alcoholic fermentation (Varela et al., 2012; Duan et al., 2015; Fairbairn et al., 2019). Therefore, TIYs differing in lipid composition may be exploited to modulate starter yeast activity (Mbuyane et al., 2021, 2022).

**Table 2:** Composition of the TIYs determined by FT-NIR.

	SC	LT	MP	SL	TD	ZF
**Total nitrogen**	8.37 ± 0.01c	8.22 ± 0.08b	8.27 ± 0.01b	8.53 ± 0.04d	7.64 ± 0.00a	8.93 ± 0.01f
**Raw protein**	52.29 ± 0.04c	51.35 ± 0.48b	51.66 ± 0.08b	53.30 ± 0.26d	47.76 ± 0.02a	55.78 ± 0.08e
**Ammonia nitrogen**	0.26 ± 0.00c	0.25 ± 0.00bc	0.22 ± 0.01a	0.25 ± 0.01b	0.28 ± 0.00d	0.23 ± 0.00a
**Saturated fatty acids (SFA)**	1.04 ± 0.00a	1.10 ± 0.02b	1.42 ± 0.01e	1.37 ± 0.01d	1.50 ± 0.00f	1.32 ± 0.00c
**Monounsaturated fatty acids (MUFA)**	2.03 ± 0.00a	3.20 ± 0.20b	3.16 ± 0.00b	4.27 ± 0.00d	6.18 ± 0.00e	3.49 ± 0.03c
**Sterols**	0.84 ± 0.00a	0.91 ± 0.01b	1.02 ± 0.00d	0.93 ± 0.00c	1.14 ± 0.00e	0.83 ± 0.00a
**Ergosterol**	0.37 ± 0.01b	0.40 ± 0.00c	0.46 ± 0.00d	0.40 ± 0.00c	0.50 ± 0.00e	0.36 ± 0.00a

SC (*S. cerevisiae*), LT (*L. thermotolerans*), MP (*M. pulcherrima*), SL (*S. ludwigii*), TD (*T. delbrueckii*), ZF (*Z. florentina*). All data are expressed as % of the dry matter. All data are reported as the average of 2 biological replicates ± standard deviation. Values with different letters (a-e) within each row are significantly different (P ≤ 0.05).

### 3.2 Polysaccharides released by TIYs

**Fig. 2:**
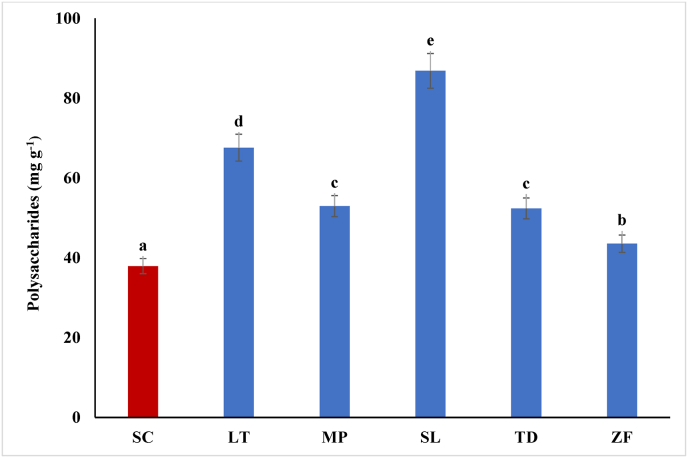
Polysaccharides released in WLS-S by TIYs (g). SC (*S. cerevisiae*), LT (*L. thermotolerans*), MP (*M. pulcherrima*), SL (*S. ludwigii*), TD (*T. delbrueckii*), ZF (*Z. florentina*). Error bars represent standard deviation of three independent experiments, each carried out in duplicate. Different letters indicate significantly different values (p ≤ 0.05).

As reported in Fig. 2, polysaccharides released by each TIY in WLS-S after 48 h, ranged from 37.9 ± 1.2 mg g^−1^ (SC-TIY) to 86.8 ± 3.4 mg g^−1^ (SL-TIY). These different amounts might depend on the different cell wall composition, and on their specific response to growth conditions (Aguilar-Uscanga and Francois, 2003). Moreover, the higher amount of polysaccharides released by non-*Saccharomyces* TIYs is in agreement with the higher release of polysaccharides during the alcoholic fermentation by active non-*Saccharomyces* yeasts (Domizio et al., 2014). The release of about 87 mg g^−1^ polysaccharides by SL-TIY seems particularly promising. In fact, 40 g hL^−1^ of SL-TIY, corresponding to the maximum IDYB recommended dose (Del Barrio-Galán et al., 2018), could release about 35 mg L^−1^ of polysaccharides, just after 48 h. Considering that *S. cerevisiae* releases on average about 150 mg L^−1^ polysaccharides during the alcoholic fermentation (Rosi et al., 2000), this addition would result in an increase in the amount of polysaccharides of about 23 %.

Moreover, it is worth highlighting that the TIYs, obtained by thermal inactivation, release mainly polysaccharides not covalently bound to the cell wall, as reported by Li and Karboune (2018). Thus, the addition of enzymes to these non-*Saccharomyces* TIYs preparations could result in a further release of polysaccharides.

**Fig. 3:**
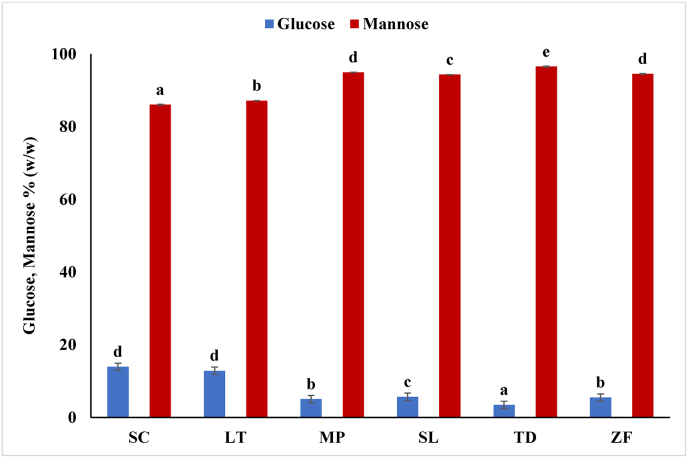
Mannose and glucose determined after acid hydrolysis of WLS-ETOH fraction. Concentrations were expressed as % on dry weight (w/w). SC *(S. cerevisiae),* LT *(L. thermotolerans),* MP *(M. pulcherrima),* SL *(S. ludwigii),* TD *(T. delbrueckii),* ZF *(Z. florentina).* Error bars represent standard deviation of three independent experiments, each carried out in duplicate. Different letters indicate significantly different values (p ≤ 0.05).

Polysaccharides composition in the WLS-ETOH fraction revealed high mannose concentrations, ranging from 86 % (SC-TIY) to 97 % (TD-TIY), and low glucose concentrations, ranging from 3 % (TD-TIY) to 14 % (SC-TIY) (Fig. 3). Notably, TIYs exhibited different mannose/glucose ratios, likely as a consequence of their different cell wall composition (Ballou, 1976; Gonçalves et al., 2002; Domizio et al., 2014, 2017).

**Fig. 4:**
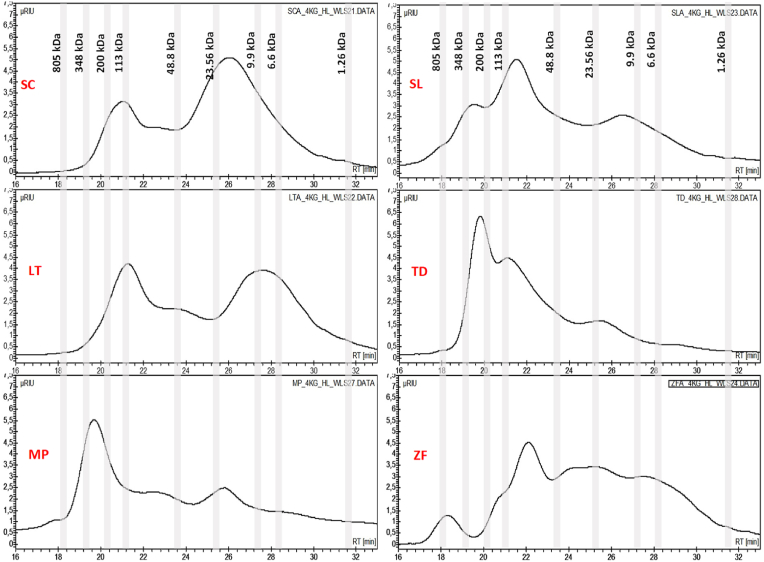
HPLC with RI detector analysis of the molecular weights profile of the polysaccharides in WLS-ETOH fraction. SC (*S. cerevisiae*), LT (*L. thermotolerans*), MP (*M. pulcherrima*), SL (*S. ludwigii*), TD (*T*. *delbrueckii*), ZF (*Z. florentina*).

The polysaccharides released by each TIY differed in the MW profiles and relevant amounts (Fig. 4), likely due to yeast biodiversity, as well as to their different growth stage at the time of the inactivation process. In particular, in comparison with the other TIYs, SC-TIY and LT-TIY showed lower amounts of polysaccharides with MW ≥ 350 kDa and similar peaks, even though with different intensities, related to polysaccharides with MW ranging from 350 kDa to ⁓100 kDa. Moreover, they both showed a second peak corresponding to polysaccharides with MW ranging from 48 kDa to 1.26 kDa and from 23 kDa to 1.26 kDa, for SC-TIY and LT-TIY, respectively. Interestingly, HPLC analyses with diode array (DAD-280 nm) detectors revealed, both for SC-TIY and LT-TIY, peaks with the highest intensity, suggesting the presence of protein residues (Supplementary Fig. 1S). These are compatible with the highest amounts of glycoproteins with MW ranging from 48 kDa to 1.2 kDa. In contrast, ZF-TIY and SL-TIY were characterized by a broad distribution of MW, ranging from 800 kDa to 1 kDa (Fig. 4). In particular, ZF-TIY was characterized by the presence of a distinctive early elution peak compatible with high MW polysaccharides (⁓800 kDa). The polysaccharides released by MP-TIY and TD-TIY showed a narrower distribution of MW, ranging mainly from 800 kDa to 48 kDa.

Polysaccharides, in particular yeast mannoproteins, significantly impact the color, astringency, and aroma of wines, depending on their compositional structure and interactions with wine matrix compounds, like polyphenols and aroma compounds. While numerous studies have highlighted mannoproteins effects on red wine color and astringency (Escot et al., 2001; Guadalupe et al., 2010; Oyón-Ardoiz et al., 2022; Manjón et al., 2021; Rinaldi et al., 2019; Vidal et al., 2003; Wang et al., 2021) fewer studies have explored their interaction mechanism with polyphenols. As reported by Li et al. (2023), competitive, associative, and precipitation mechanisms modulate astringency (Li et al., 2023), and differences in polysaccharide structure and phosphorylation degree influence their interaction with polyphenols, affecting wine color and astringency (Nguela et al., 2023). Accessibility of Malvidin-3-O-Glucoside to the negatively charged mannosyl-phosphate groups affects color intensity (Bicca et al., 2022). The molecular weight of mannoproteins is crucial for red wine color stability. High molecular weight mannoproteins harboring a high number of binding sites might overreact with polyphenols, leading to flocculation and reduction of anthocyanin and other phenolic compounds, compromising color stability (Li et al., 2023). Polysaccharides molecular weight and composition affect interactions with volatile compounds. Their conformational and compositional structure, particularly the proteic component, plays a vital role in retaining aromatic compounds, driven by hydrophobic interactions (Chalier et al., 2007). Mannoproteins have been reported to reduce visible protein haze in white wine, with their effectiveness influenced by the mannose-to-glucose ratio (Ribeiro et al., 2014), where higher mannose proportion enhances protein stability.

Thus, although the actual impact of polysaccharides on different wines needs to be further elucidated, the biodiversity of non-*Saccharomyces* inactivated yeast derivatives in terms of polysaccharides amounts, composition and MW, could be functional to the achievement of different oenological objectives.

### 3.3 Proteins released by TIYs in WLS-S

Protein mixtures released in WLS-S by TIYs, after TCA precipitation, were characterized by nHPLC-MS/MS-based shotgun proteomics. The spectra obtained were searched against the UniProtKB database, taxonomically restricted to *Microorganisms*. The majority of the protein entries (912 unfiltered gene products, false discovery rate <1%) were inventoried for TIY from *S. cerevisiae* (SC), whose genome and proteome are completely sequenced. The search was also carried out interrogating the curated SwissProt *Fungi* database with the Mascot search engine, to obtain an indicative ranking of the protein abundances through the emPAI values (Ishihama et al., 2005). The protein identifications in the UniprotKB and curated SwissProt databases are listed individually for the six yeast species investigated in the Supplementary Tables S1 and S2, respectively. Due to the incomplete annotation of the genomic and proteomic databases for the non-*Saccharomyces* species, many proteins were identified by homology.

**Fig. 5:**
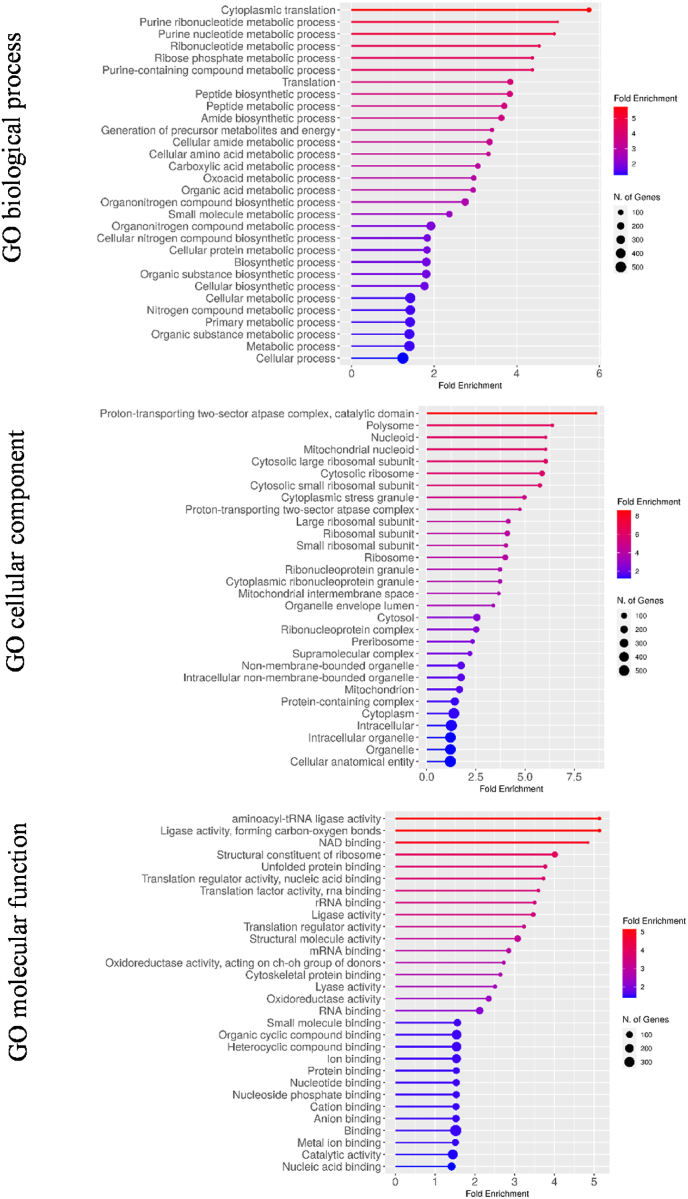
GO functional analysis of the expression proteome for *S*. *cerevisiae*, according to Biological process, Cellular component and Molecular function. GO enrichement and analysis were performed using the open source ShinyGO v0.75 tool.

A functional Gene Ontology (GO) analysis of the expression proteome demonstrated that the enriched gene products of *S. cerevisiae* involved biological processes such as translation, amino acid biosynthesis and metabolism, biosynthesis of organic acid and peptide while, as concerns the cell localization they were predominantly cytoplasmic and ribosomal enzymes (Fig. 5). Only 9 proteins, including glycolytic enzymes (glyceraldehyde-3-phosphate dehydrogenase, pyruvate kinase, enolase, fructose-bisphosphate aldolase, phosphoglycerate kinase), ribosomal proteins (40S ribosomal protein, 60S ribosomal protein) and other cytosolic and/or plasma membrane proteins (plasma membrane ATPase, elongation factor 1-alpha) were common to all the TIYs. Other enzymes involved in glycolysis, alcoholic fermentation, pentose phosphate pathway (glucose 6-phosphate isomerase, pyruvate decarboxylase, triose phosphate isomerase, alcohol dehydrogenase, transaldolase) and a variety of heat shock proteins were found in the majority of the TIYs. The comparison of the set of proteins released by SC*-*TIY with those of non-*Saccharomyces* TIYs, considered as a whole, revealed that 91 proteins were shared by the two groups. These included mainly proteins involved in gluconeogenesis, glycolysis, protein folding and translational elongation. On the other side, 316 proteins were unique to SC-TIY, while 68 proteins appeared specific to the non-*Saccharomyces* TIYs. Of these, in addition to alcohol dehydrogenase that was common to all non-*Saccharomyces* TIYs, 17 proteins were exclusive for ZF-TIY, 13 for SL-TIY, 8 for MP-TIY, 3 for LT-TIY and 3 for TD-TIY (Supplementary Table S2). The predominant gene products identified for non-*Saccharomyces* TIYs appeared functionally homologous to those of SC-TIY. Based on the emPAI values, the most represented proteins were glycolytic and ribosomal enzymes, redox modulators, and heat shock proteins in all the TIY samples. The significantly lower number of proteins that were cataloged for non-*Saccharomyces* yeasts TIYs (121 ZF-TIY, 72 TD-TIY, 120 SL-TIY, 75 MP-TIY), and the apparent discrepancy among the protein sets obtained for the different TIYs, should be interpreted as a consequence of the poor database annotation for non-*Saccharomyces* yeasts rather than reflecting an actual metabolic divergence. Therefore, the qualitative and quantitative proteomic profiles for TIYs of non-*Saccharomyces* yeasts depict a partial portrait that could be implemented with the forthcoming database compilation. The abundance of cytosolic proteins was, in part, expected due to cell disruption following inactivation. These proteins largely exceeded the presence of proteins properly classifiable as the “secretome”. Interestingly, cell wall and cell wall associated proteins, also including cell wall mannoproteins which have technological significance in wine, were identified for several yeast species. However, their presence is likely underestimated because the utilization of WLS for resuspending TIYs could induce the loss of poorly soluble proteins such as membrane or membrane associated proteins. Furthermore, the MS-based identification of highly glycosylated proteins could be intrinsically challenging, although a preliminary enzymatic N-deglycosylation was attempted to maximize the coverage of these protein compounds. Further studies are needed to understand the impact of the proteins released by different TIYs both on starter yeasts fermentative performances and quality of the final product.

### 3.4 TIYs proteins recovered from WLS-ETOH

**Fig. 6:**
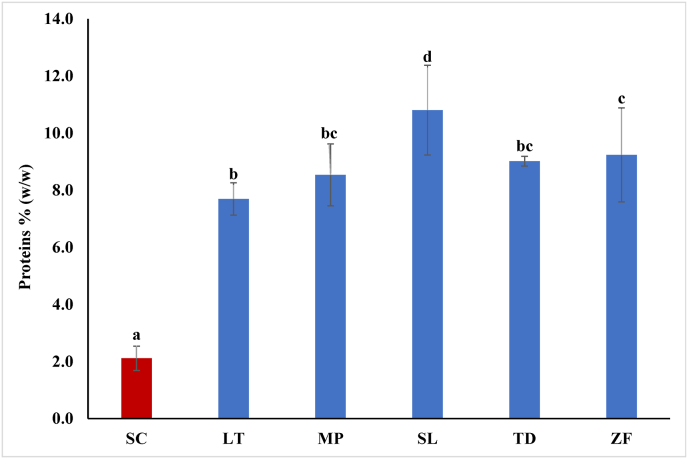
Protein content (%) of the polysaccharides recovered from WLS-ETOH for each TIY. SC (*S. cerevisiae*), LT (*L. thermotolerans*), MP (*M. pulcherrima*), SL (*S. ludwigii*), TD (*T. delbrueckii*), ZF (*Z. florentina*). Error bars represent standard deviation of three independent experiments, each carried out in duplicate. Different letters indicate significantly different values (p ≤ 0.05).

By comparing different methods for yeast cell disruption, Bzducha-Wróbel et al. (2014) found that autoclaving was characterized by the lowest level of protein solubilization, indicative of the poorest effectiveness of yeast cell disruption. Accordingly, protein recovered from WLS-ETOH was around 2 % for SC-TIY. Instead, all the non-*Saccharomyces* TIYs showed a higher percentage of proteins, ranging from 8 to 11 % (Fig. 6).

To improve the detection of glycosylated cell wall proteins among those released in WLS-ETOH, selected bands containing high MW proteins (⁓250 kDa), detected with Schiff reagent, were excised from SDS PAGE gel (Supplementary Fig. S2) and analyzed individually by nHPLC-MS/MS, after sequential N-deglycosylation with PNGase F and trypsinolysis. Proteins identified in the high molecular weight gel bands are reported in Supplementary Table S3. In this way, the presence of the cell wall mannoproteins Pir1 in LT-TIY, MP-TIY, SL-TIY, ZF-TIY, and Hsp150 in SC-TIY was demonstrated in the very high MW fraction of proteins that did not enter the SDS-PAGE gel. Moreover, all TIYs but SL-TIY, released a Clp R domain-containing protein. Clp proteins are proteolytic enzymes that use ATPases associated with diverse cellular active (AAA+) domains to unfold proteins for degradation. They work as molecular chaperones and energy-dependent proteases and are implicated in a variety of cellular processes including sporulation, DNA replication, protein turnover, stress tolerance and acclimation, and regulation of gene expression (Porankiewicz et al., 1999).

### 3.5 TIYs antioxidant activity, sulphur compounds and oxygen consumption

IDYB antioxidant activity has been related to the presence of reduced glutathione (GSH) (Romanet et al., 2019), thiols (Gallardo-Chacón et al., 2010), lipids (Fornairon-Bonnefond et al., 2003; Nioi et al., 2022) and neutral polysaccharides (Jaehrig et al., 2007). Here, to evaluate the antioxidant activity of the different TIYs, DPPH assay was carried out. Results indicate that SC-TIY and LT-TIY display the highest and lowest antioxidant activities, respectively (Table 3). Total thiols and GSH were evaluated to gather further information on TIYs released antioxidant molecules. Significant differences (p < 0.05) in the amount of thiols released were found for SC-TIY and SL-TIY (5.158 ± 0.021 mg g^−1^ and 4.119 ± 0.020 mg g^−1^, respectively). SC-TIY also released the highest amount of GSH (1.128 ± 0.181 mg g^−1^). This was significantly higher (p < 0.05) than that found for the remaining non-*Saccharomyces* TIYs, ranging from 0.104 ± 0.029 mg g^−1^ (LT-TIY) to 0.852 ± 0.095 mg g^−1^ (MP-TIY) (Table 3).

**Table 3:** Antioxidant activity (DPPH), total thiols and reduced glutathione (GSH) of TIYs.

Sample	DPPH mmol g^−1^	Total Thiols mg g^−1^	GSH mg g^−1^
**SC**	1.392 ± 0.005 f	5.158 ± 0.021 e	1.128 ± 0.181 e
**LT**	0.888 ± 0.002 a	4.644 ± 0.012 c	0.104 ± 0.029 a
MP	1.103 ± 0.012 d	4.239 ± 0.047 b	0.852 ± 0.095 d
**SL**	1.054 ± 0.011 c	4.119 ± 0.020 a	0.625 ± 0.086 c
**TD**	1.236 ± 0.013 e	4.950 ± 0.023 d	0.636 ± 0.142 c
**ZF**	1.018 ± 0.014 b	4.262 ± 0.051 b	0.415 ± 0.053 b

DPPH activity is expressed as mmol of trolox per g of TIY; total thiols are expressed as mg of GSH per g of TIY and GSH as mg of GSH per g or TIY. SC (*S. cerevisiae*), LT (*L. thermotolerans*), MP (*M. pulcherrima*), SL (*S. ludwigii*), TD (*T. delbrueckii*), ZF (*Z. florentina*). All data are reported as the average of 3 replicates ± standard deviation. Values with different letters (a-f) within each column are significantly different (P ≤ 0.05).

Andújar-Ortiz et al. (2012) found that a yeast derivative, not enriched with GSH, was able to release 0.46 mg L^−1^ GSH (approximately 1.53 mg g^−1^), which is close to the amount released by SC-TIY (1.13 mg g^−1^) and higher that those of non-*Saccharomyces* TIYs (Table 3). This difference in GSH content may be attributed to the specific properties of the yeast species used to produce the TIYs. Additionally, the manufacturing process, particularly the application of high temperatures, could affect the GSH concentration. High temperatures might degrade GSH or lead to its consumption in Maillard reactions with reducing sugars, contributing to its reduction in the final inactive dry yeast product (Lee et al., 2010).

**Fig. 7:**
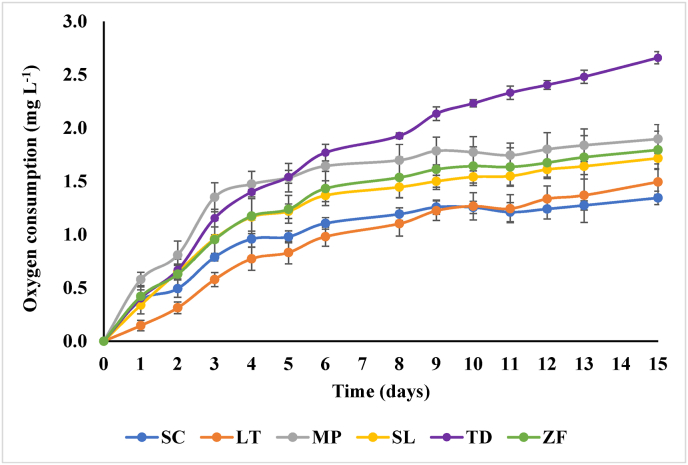
TIYs oxygen consumption. SC (*S. cerevisiae*), LT (*L. thermotolerans*), MP (*M. pulcherrima*), SL (*S. ludwigii*), TD (*T. delbrueckii*), ZF (*Z. florentina*). Error bars represent standard deviation of three independent experiments, each carried out in duplicate.

TIYs kinetics of oxygen consumption was measured in an iron and copper enriched wine like solution (E-WLS), saturated with oxygen, as reported by Pons-Mercadé et al. (2021). Fig. 7 shows the oxygen consumption over 15 days. Oxygen consumption rate (OCR t0) and total oxygen consumption capacity (TOCC) were determined by applying the kinetics model proposed by Pascual et al. (2017).

TIYs harbored significantly different OCR t0 (Table 4) with MP-TIY showing the highest value (0.84 ± 0.01 mg L^−1^ day^−1^). Interestingly, SC-TIY, although releasing the highest amount of thiols and GSH, was characterized by the lowest TOCC (1.65 ± 0.14 mg L^−1^). Among the non-*Saccharomyces*, TD-TIY showed the highest TOCC value (4.06 ± 0.13 mg L^−1^) (Table 4). OCRt0 and TOCC values of the non-*Saccharomyces* TIYs were similar or even higher than those found by Pons-Mercadé et al. (2021) for two strains of *S. cerevisiae,* specifically selected for their ability to consume oxygen and evaluated under the same experimental conditions.

**Table 4:** Oxygen consumption rate at time 0 (OCR t0) and total oxygen consumption capacity (TOCC) of TIYs.

TIY	OCR t0 (mg L^−1^ day^−1^)	TOCC (mg L^−1^)
SC	0.50 ± 0.03b	1.65 ± 0.14a
**LT**	0.28 ± 0.03a	2.44 ± 0.38c
**MP**	0.84 ± 0.01d	2.43 ± 0.07c
**SL**	0.52 ± 0.03bc	2.27 ± 0.20b
**TD**	0.51 ± 0.01b	4.06 ± 0.13d
**ZF**	0.59 ± 0.07c	2.23 ± 0.26b

SC (*S. cerevisiae*), LT (*L. thermotolerans*), MP (*M. pulcherrima*), SL (*S. ludwigii*), TD (*T. delbrueckii*), ZF (*Z. florentina*). All data are expressed as the average of 3 replicates ± standard deviation.Values with different letters (a-d) within each column are significantly different (P ≤ 0.05).

**Fig. 8:**
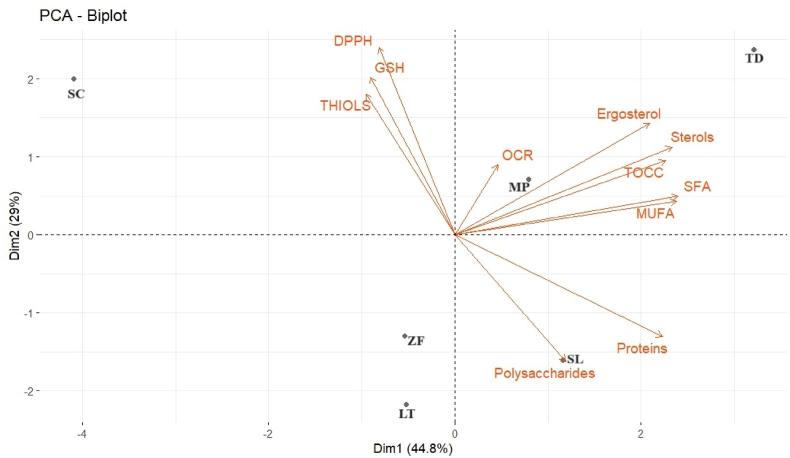
Principal component biplot graph. Vectors represent variable (mean values). Scores represent TIY. SC (*S. cerevisiae*), LT (*L. thermotolerans*), MP (*M. pulcherrima*), SL (*S. ludwigii*), TD (*T. delbrueckii*), ZF (*Z. florentina*).

Aiming to highlight the relationships among the variables (shown as vectors) and TIYs (shown as scores), a principal component analysis (PCA) was performed (Fig. 8). The two principal components (PCs) accounted for 73.8 % of the total variance. Based on this analysis, oxygen consumption seems likely to depend more on lipids content than on the reducing compounds. In MP-TIY and TD-TIY, that harbor significantly higher amounts of ergosterol, the higher oxygen consumption might be due to the two double bonds in the B-ring of ergosterol conferring antioxidant properties. Other authors claimed that oxygen consumption might be also due to the content of unsaturated lipids (Fornairon-Bonnefond et al., 2003; Nioi et al., 2022). Accordingly, SC-TIY shows the lowest MUFAs content. On the other hand, the model shows that oxygen consumption is not related to the antioxidant activity resulting from the DPPH assay which measures the free radical scavenging capacity. Accordingly, SC-TIY was characterized by the lowest oxygen consumption despite the highest DPPH and GSH values. In agreement, inactivated dry yeasts rich in GSH react faster with oxygen, but their TOCC is not correlated with their GSH content (Bahut et al., 2020; Pons-Mercadé et al., 2021). SL-TIY, characterized by the highest amount of polysaccharides, mainly corresponding to mannoproteins, is located on the positive side of PC1 suggesting that also mannoproteins are involved in oxygen consumption, even though to a lower extent compared to the other compounds analyzed.

Hence, not only soluble compounds, but also the insoluble fraction still present in the cell wall, such as mannoproteins (Jaehrig et al., 2008) and lipids (Nioi et al., 2022) appear to be involved in oxygen consumption.

## 4 Conclusion

Qualitative and quantitative analyses were carried out in WLS to characterize TIYs originating from different yeast species. The results obtained collectively emphasized the significant biodiversity among these TIYs, revealing variations in the content and composition of proteins, polysaccharides, and lipids. While the influence of the analyzed compounds on wine stability and sensory properties has already been largely discussed, the impact of the different TIYs on the overall sensory and stability attributes of wine needs to be assessed under real winemaking conditions. Furthermore, the observed biodiversity of TIYs, particularly in terms of oxygen consumption, opens new scenarios on the exploitation of non-*Saccharomyces* yeasts derivatives.

## Author contributions

Conceptualization P.D.

Data curation, P.D., I.M., V.C., G.P., F.C.

Formal analysis, P.D., V.C.; I.M.

Funding acquisition P.D.

Investigation, V.C., F.C., G.P., E.T., M.B.

Methodology, P.D.

Resources, P.D., F.C., G.P.

Supervision, P. D, I.M.

Visualization, P. D, I.M.

Writing - original draft, V.C.,G.P, F.C.

Writing - review & editing, P.D., I.M.

## Declaration of competing interest

Relating to the manuscript entitled “Non-Saccharomyces yeast derivatives: characterization of novel potential bio-adjuvants for the winemaking process” submitted for inclusion in “Current Research in Food Science”, I state that there are no financial conflicts of interest to disclose.

## Data Availability

No data was used for the research described in the article.
