# Supplementary material for: Non-Saccharomyces yeast derivatives: Characterization of novel potential bio-adjuvants for the winemaking process
Source: Curr Res Food Sci. 2024 May 22;8:100774. doi: 10.1016/j.crfs.2024.100774 (PMC11153934; doi:10.1016/j.crfs.2024.100774)
Supplement: Multimedia component 1 [file mmc1.docx]

**Supplementary Figure S1**


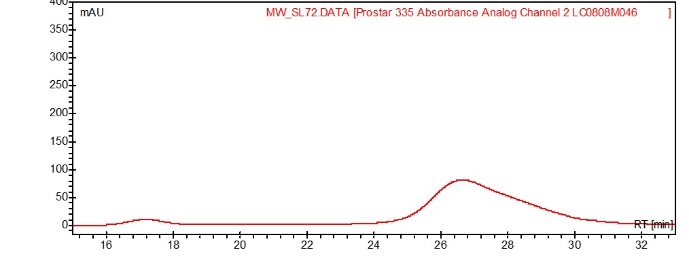

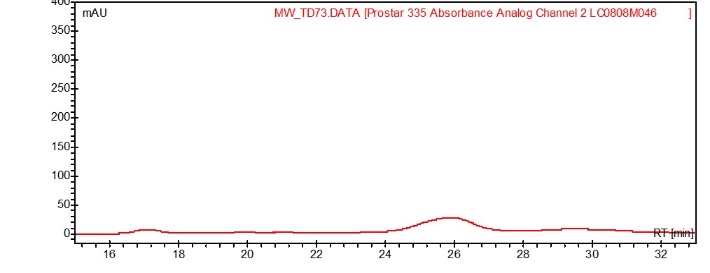

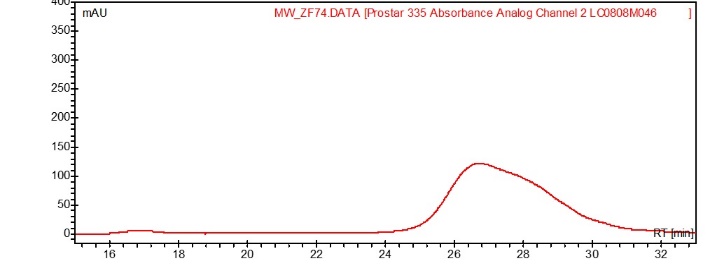

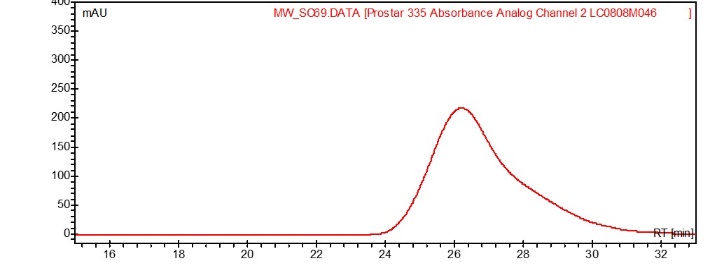

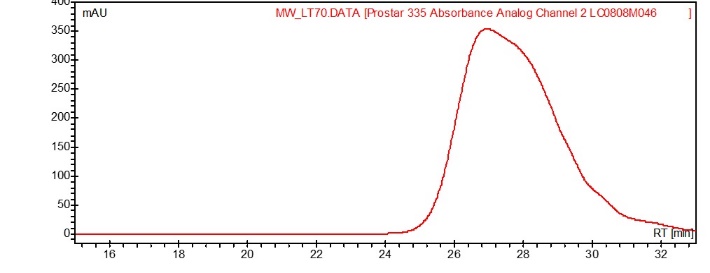

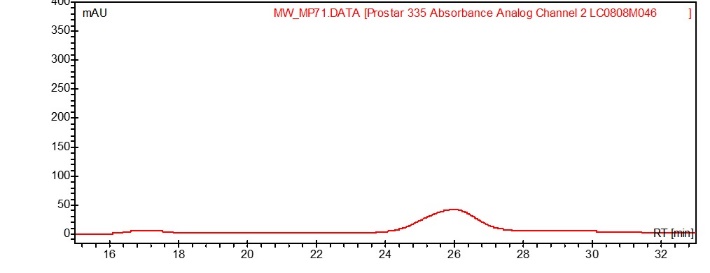


**805 kDa**

**348 kDa**

**200 kDa**

**113 kDa**

**48.8 kDa**

**23.6 kDa**

**9.9 kDa**

**6.6 kDa**

**1.26 kDa**

**805 kDa**

**348 kDa**

**200 kDa**

**113 kDa**

**48.8 kDa**

**23.6 kDa**

**9.9 kDa**

**6.6 kDa**

**1.26 kDa**

**SC**

**LT**

**MP**

**SL**

**TD**

**ZF**
