# Supplementary figures and images for: Non-Saccharomyces yeast derivatives: Characterization of novel potential bio-adjuvants for the winemaking process
Source: Curr Res Food Sci. 2024 May 22;8:100774. doi: 10.1016/j.crfs.2024.100774 (PMC11153934; doi:10.1016/j.crfs.2024.100774)

**Supplementary Figure S3**


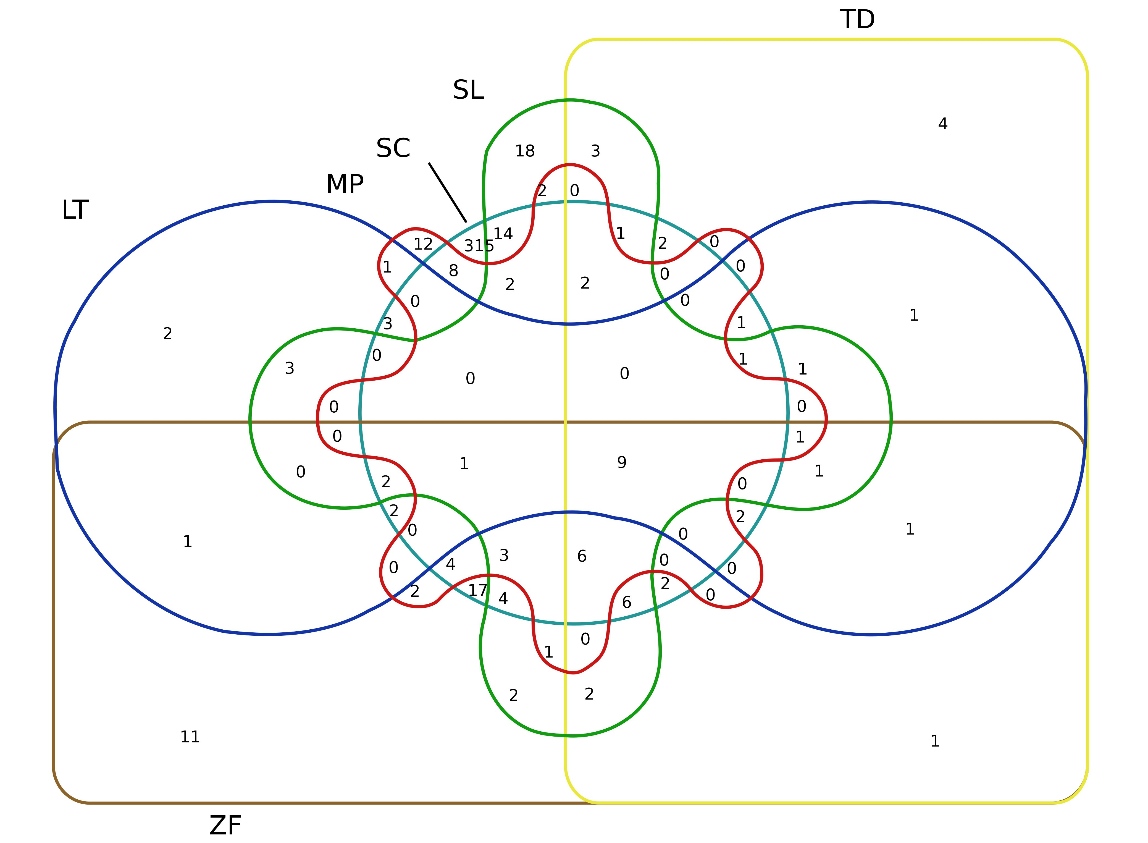

Supplement: Multimedia component 2 [file mmc2.docx]
